# Nutrition and Dietary Patterns: Effects on Brain Function

**DOI:** 10.3390/nu17071169

**Published:** 2025-03-28

**Authors:** Roser Granero, Gemma Guillazo-Blanch

**Affiliations:** Departament de Psicobiologia i Metodologia de les Ciències de la Salut, Institut de Neurociències, Universitat Autònoma de Barcelona, 08193 Barcelona, Spain; gemma.guillazo@uab.cat

## 1. Introduction

Dietary habits are strongly associated with neuropsychological functioning. Etiological studies suggest that healthy cardiometabolic status is a protective factor against vascular dementia [1] and neurological disease [2], and balanced nutrition may slow the rate of cognitive decline with age [3]. These associations are observed throughout life. First, poor dietary quality during the fetal and early postnatal period negatively impacts neurodevelopment in early life (especially in cases of deficiencies in essential nutrients and protein-energy malnutrition) [4]. Second, dietary patterns in early childhood have been associated with differences in brain morphology, neurodevelopment, and cognitive performance [5]. Long-term longitudinal studies have also linked dietary patterns in childhood to posterior cognitive performance during pre-adolescence [6]. Third, studies on the elderly (in the general population, hospital settings, or long-stay units) have observed that nutritional status has a significant influence on vital and functional prognosis and that a balanced diet is associated with better performance in tests assessing memory, attention, reasoning skills and other executive functions [7]. Studies of the impact of diet and nutrition on age-related cognitive decline are a growing field.

In recent years, nutrition research has increasingly emphasized the major impact of sociocultural factors on dietary practices, including preferences for specific foods, selective eating, and different cooking styles. For example, the Western diet is characterized by large amounts of saturated fats, refined sugars, and processed foods and has been linked to impaired learning and memory [8]. Conversely, dietary patterns like those typical of the Mediterranean [9,10,11] and Nordic countries [12] may be important protective factors for cognitive health. Etiological research suggests that the potential of these diets for brain function may be mediated by their particular anti-inflammatory, lipid-lowering, antioxidant, gut–brain-axis modulating, and ligand activities in cell signaling pathways.

However, while most studies have observed a strong relationship between nutritional habits, eating behaviors, and biopsychosocial dysfunction, this has not always been the case. The theoretical models used in these studies may explain some of these discrepancies. For instance, the classical model of homeostatic feeding posits that individuals ingest food when they perceive that their energy resources are depleted and stop eating (or abstain from) when those energy levels are restored. This traditional theory does not account for the implications of psychosocial functioning. Current models of eating behavior are substantially more complex, encompassing a broader range of constructs, including homeostatic balance, eating habits, food choices/motives, nutrition practices, and dieting. According to these newer models, eating behavior and dietary decision-making are driven by complex interactions between various motivational, affective, and social factors, including the availability of certain foodstuffs, sociocultural contexts, cognitive influences, and internal emotional states such as boredom and stress. Plassmann and colleagues present an interdisciplinary framework for dietary decision-making in which three broad systems interact [13]: (a) the physiology of homeostatic drivers, which regulate eating based on energy needs and the availability of specific foods; (b) the neuroeconomics of dietary decision-making, which is regulated by the connections between the central nervous system and emotions and environmental variables; and (c) the emerging research on the role of the gut microbiome in reward-based dietary decision-making, which has attracted so much interest in the fields of nutrition and neurology. It is known that the combination of bacteria and other microorganisms residing in the gut makes a major contribution to host health through the mediation of homeostatic and reward processes [14,15]. Scientific advances in sequencing and bioinformatics have enabled a more accurate study of communication mechanisms between the microbiome, brain, and behavior [16].

This Special Issue provides evidence for the complex relationships between nutrition and multiple biopsychosocial dysfunctions and the neurobiological basis of these interactions. The study by Arata and colleagues specifically focuses on the underlying mechanisms of the effects of androgens on body weight and appetite in females, considering the role of endogenous oxytocin (OT) (contribution 1). This peptide hormone and neuropeptide is produced in the hypothalamus, is released by the posterior pituitary, and is involved in several processes (including lactation, parturition, mother-infant interaction, psychosocial function, and regulation of eating behaviors) [17]. Arata et al.’s study consisted of a controlled assay on rats and showed that the effects of androgens on OT may be affected by the estrogen milieu and that elevated OT levels may blunt the appetite and help to prevent obesity under estrogen-deficient conditions.

Other areas of particular interest explored in this Special Issue include the relationship between nutrition and neurocognition in eating disorders (EDs) and other physical and psychiatric conditions. Special attention is given to the underlying processes of dietary habits that support the maintenance and progression of cognitive function.

## 2. Nutrition, Neurocognition and ED

It is well known that persistent disturbances in eating behavior can lead to the onset and progression of several mental illnesses, which can increase the likelihood of comorbid cardiometabolic disorders. Several common biological pathways have been identified that explain these comorbidities between poor-quality diets and mental disorders, including oxidative stress, hypothalamic–pituitary–adrenal axis dysregulation, and regulation of monoamine and overall neuronal function [18,19]. EDs are mental conditions strongly associated with alterations in nutrition and eating habits. Epidemiological and clinical research show that anorexia nervosa, bulimia nervosa, and binge eating have a significant impact on personal well-being, including physical disturbances (gastrointestinal, renal, and cardiovascular problems), psychological distress (mood and anxiety disorders) and social issues (loss of relationships and isolation). Compared to healthy control groups, eating disorder samples exhibit specific brain markers, including impaired neurocognitive processes, altered functional connectivity, and even different brain structures. Moreover, specific impairments in executive functions, sensitivity to reward and punishment, and neurobiological mechanisms (disturbances in neurotransmitter functions such as dopamine, serotonin, and endogenous opiates) have also been related to the onset and persistence of eating behavior-related problems.

This Special Issue includes studies providing new evidence for the complex relationship between nutrition patterns, EDs, and brain function. First, Krug et al. (contribution 2) present a critical narrative review of multiple neurocognitive findings from experimental work on the most prevalent ED subtypes. The neuropsychological processes covered by their study include cognitive flexibility, central coherence, attentional bias, reward processing, inhibitory control, emotion regulation, and impulsivity. The ED subtypes examined in their study include anorexia nervosa, bulimia nervosa, and binge eating, among others. Cognitive functioning among overweight individuals was also reviewed and analyzed. The authors conclude that some neuropsychological processes seem to be transdiagnostic across multiple EDs and weight issues, such as impulsivity, lack of cognitive flexibility, emotion management difficulties, and impaired reward processing. In contrast, other such processes seem more specific to certain ED subtypes, such as an enhanced focus on detail in anorexia nervosa and heightened attention bias toward food-related stimuli in bulimia nervosa and binge eating. This review covers the latest scientific evidence on neurocognitive factors across the most prevalent ED subtypes, thus contributing to developing more nuanced therapeutic approaches that address both the unique (specific) and shared (transdiagnostic) neurocognitive patterns driving disordered eating behaviors.

The second study included in this Special Issue, published by Seelarbokus and colleagues (contribution 3), explores the prevalence of food addiction in stroke patients, as well as its association with the main risk factors of vascular stroke (dyslipidemia, hypertension, diabetes, and obesity). Food addiction is commonly characterized by the persistent and uncontrollable over-consumption of (hyper)palatable foods despite the adverse consequences (such as obesity and increased cardiovascular risk). The term “addiction” is based on neuro-biological evidence suggesting that certain components of (hyper)-palatable foods (salt, sugars, and fats) may induce addictive behaviors by activating dopamine reward systems in the brain [20]. Food addiction is not included as an eating disorder in the principal diagnostic taxonomies, but it has been strongly associated with disordered eating. Seelarbokus et al. analyzed data gathered from N = 101 patients who were consecutively hospitalized for a first stroke event in a neurovascular unit (mean age of 62.8, 61 men and 40 women). Based on a self-report questionnaire, the prevalence of participants who were screened positive for food addiction was 5%, while 38.6% reported at least one symptom. The most strongly related vascular risk factor with food addiction was dyslipidemia, followed by diabetes. The authors note the importance of administering standardized measurement tools to identify the presence of food addiction (not only as a specific diagnosis but also as the key symptom criteria) during the routine evaluation of stroke patients. They suggest that treatment units should include multidisciplinary teams of neurologists, dietitians, and psychiatrists/psychologists specialized in disordered eating in order to develop precise treatment strategies, particularly considering that stroke is ranked as the second leading cause of death in the Global Burden of Disease.

The third study in this Special Issue was conducted by Ciancarelli and colleagues (contribution 4) and focused on stroke patients. Their systematic review provides cumulative evidence of the benefits of neuro-nutrition as an adjuvant strategy in personalized nutritional interventions aimed at improving post-stroke neuroplasticity and neurorehabilitation. The review identifies nutrients and dietary patterns supporting recovery by mitigating oxidative stress, inflammation, and gut–brain axis disturbance. Special attention is paid to the role of minerals in oxidative/nitrosative stress, the effects of antioxidant and anti-inflammatory diets (such as Mediterranean and Ketogenic), the description of stroke-induced modifications of the gut-bran axis and dysbiosis, and gut microbiota as a potential target for post-stroke functional recovery.

Reivan-Ortiz et al. (contribution 5) explore the underlying mechanisms and mediating links contributing to body mass index (BMI) and eating disorder (ED) severity based on a large set of variables, including emotion regulation capacity, impulsiveness, anxiety and stress levels, and decision-making performance. Their study examines a broad spectrum of ED subtypes, including bulimia nervosa, binge eating, night eating syndrome, and other specified feeding and ED. Path analysis revealed that BMI was directly associated with ED severity and that ED symptomatology mediated the relationship between BMI and emotion regulation strategies, impulsivity, reasoning skills, and stress levels. The authors conclude that the structural coefficients were invariant across sexes, but differences were identified based on ED subtypes. These findings highlight the important role of nutrition education in managing food-related anxiety and fears and in preventing the onset and progression of ED.

Estévez et al. (contribution 6) carried out a study among a large population-based sample (N = 1076 men and women aged between 18 and 61) to test the mediating role of coping strategies (including emotion regulation capacity) in the relationship between metacognition, impulsivity, and EDs. The authors confirm that coping partially mediates the associations between executive functioning and ED severity. One strength of this study is its focus on metacognition. This concept refers to beliefs, awareness, understanding, and interpretation regarding one’s thought processes, which have been defined as central targets in metacognitive therapy, having been strongly associated with a greater likelihood of multiple psychopathologies and negative emotions (including anxiety, depression, and ED). The findings of this research support the use of metacognitive therapy (a third-wave cognitive therapeutic approach aimed at improving ED outcomes by modifying metacognitive beliefs that perpetuate states of selective attention fixation, rumination, and worry). Previous studies have also proposed an integrated cognitive behavioral therapy complemented with metacognitive therapy for treating different ED subtypes [21,22].

This Special Issue also includes two systematic reviews of the effect of Transcranial Direct Current Stimulation (tDCS) on two ED types, namely anorexia nervosa (contribution 7) and binge eating (contribution 8). tDCS is a non-invasive technology that is currently used in many areas of medicine for the treatment of various neurological and psychiatric disorders (including EDs). The procedure is based on stimulation of the cerebral cortex using low-intensity electrical currents by means of sponge electrodes with opposite polarities (anode and cathode) soaked in saline and applied to the scalp. Sessions typically last between 15 and 30 min. The capacity of tDCS for modulating cortical activity in humans has been extensively studied, as has its impact on perceptual, cognitive, and behavioral functions. However, the number of studies in the field of EDs is limited. The two reviews included here summarize the evidence on how tDCS can influence neurofunctional reorganization and behavioral changes by altering cortical excitability in anorexia nervosa and binge eating. They conclude that tDCS is well-suited for neuromodulatory treatment of EDs as it helps to reduce cognitive biases related to eating (such as body dissatisfaction and drive for thinness), and also to improve eating practices, modify food intake and enhance body composition (especially weight recovery in anorexia nervosa). Interestingly, tDCS has also been proven to be effective in reducing depression and anxiety levels and for increasing self-esteem and global psychological adjustment.

## 3. Diet, Cognition and Bio-Psychological Illness

Other studies in this Special Issue focus on how eating patterns contribute to physical and neuropsychological functioning among diverse populations. Maury and colleagues (contribution 9) assessed serum homocysteine and inflammatory cytokines (IFN-γ, IL-6, IL-1β, TNF-α) as potential biomarkers for alleviating depression among veterans with Gulf War Illness (also called Gulf War Syndrome). This illness, of debated etiology and pathophysiology, is a complex neuropsychological condition characterized by chronic pain and fatigue, hypertension, insomnia, cognitive impairment (such as reduced coordination and memory problems), and negative mood symptoms. Post-traumatic stress disorder and depression are two highly probable comorbid disorders in relation to Gulf War Illness [23]. Maury et al. analyzed data recruited from N = 33 veterans who met clinical criteria for the syndrome (11 women and 22 men, mean age ~ 54 yrs). The findings from a clinical trial showed that after a one-month dietary plan aimed at reducing excitotoxicity and increasing micronutrients, more than half of the participants reported a significant reduction in depression symptom levels. The authors thus conclude that serum homocysteine and cytokine IFN-γ may serve as possible biomarkers for alleviating depression using a low-glutamate diet that could complement effective pharmacological and psychological therapies [24].

Tsuji and colleagues (contribution 10) assessed the role of L-carnosine in CD157 knockout (KO) mice, a murine model for autism spectrum disorder (ASD). Carnosine is a dipeptide molecule composed of the amino acid beta-alanine and histidine, which is highly concentrated in the muscles, the gastrointestinal tract, and the brain. It is commercially available as a nutritional supplement for recovery from fatigue, given its potential antioxidant, anti-inflammatory, and neuroprotective properties, improving brain functioning and protecting the central nervous system. Animal (rodent) models have indicated that L-carnosine may be involved in stress-induced corticosterone responses and anxiety behaviors. Tsuji et al.’s findings are consistent with this hypothesis and have interesting implications for psychiatry, as this molecule may relieve anxiety by suppressing stress-induced hyperresponsivity in a subgroup of individuals with ASD. Previous research has also observed that L-carnosine may be deficient among children with ASD [25,26], but results demonstrating its effectiveness in this regard are inconsistent [27].

Talik and colleagues employed a double-blind placebo-controlled methodology to study the effects of guarana on cognitive performance, motivation, mental energy, mental fatigue, mood parameters, and the effect of this component and the role of sympathetic activation (contribution 11). Guarana is a climbing plant in the Sapindaceae family native to the Amazon basin. It is typically used as a dietary supplement or herb due to the stimulant properties of its seeds [28]. Previous studies have found that guarana seeds may enhance cognitive performance (alertness, vigilance, reaction time, and attention) [29] among the general population ([30] and within clinical samples of patients with fatigue and pain symptoms [31]. Guarana also has anti-inflammatory, antioxidant, and anti-aging effects [32] and may promote wound healing [33]. Studies on the benefits of guarana for psychological well-being, anxiety, and mood have also yielded promising results [34].

Lacasa et al. analyzed the benefits of beta-glucan on fatigue, unrefreshing sleep, anxiety and depression symptoms, and health-related quality of life in patients diagnosed with myalgic encephalomyelitis (also called chronic fatigue syndrome) by means of a randomized double blind placebo-controlled trial (contribution 12). This syndrome is a serious, long-lasting illness causing widespread pain, physical and mental exhaustion, and severe fatigue that prevents individuals from performing typical everyday activities [35]. It has been defined as a complex neurological disease with widely varying recovery rates [36,37]. In a sample of N = 67 patients consecutively recruited from a single outpatient tertiary referral center, Lacasa and colleagues observed that beta-glucan helped to reduce cognitive fatigue symptoms, with major beneficial effects all along the gut microbiota–immune–brain axis. Interestingly, the authors suggest that this positive effect could be extended to a disease model of cognitive decline.

This Special Issue also includes a review by Daida and colleagues (contribution 13) on the role of diet and energy perturbations in the emergence of neurodevelopmental and neurodegenerative disorders mediated by isoforms of glucose transporters, concretely GLUT3 (a protein that in humans is encoded by the SLC2A3 gene). GLUT3 is mainly found in the brain and is responsible for transporting glucose across the plasma membranes of mammalian cells (it was originally designated as the neural glucose transporter [GLUT]). In the first part of the study, Daida et al. detail the expression and function of GLUT3 in human and animal models, after which they describe how maternal diet and genetic modifications of GLUT3 influence the propensity to develop neurodevelopmental disorders. This study contributes to current systematic reviews exploring the role of GLUT3 as a biomarker to aid diagnosis and prognostication of diverse severe physical conditions, such as carcinoma [38,39,40,41] and cardiomyopathies [42], as well as other reviews on the role of GLUT3 in psychopathological conditions such as depression [43] and Alzheimer’s disease [44].

A final area of special scientific interest is the role of nutrition in the neurological functioning of professional athletes. Since peak sports performance relies on adequate cognitive functions [45], nutritional habits are crucial. The high demands of professional sports have led to research on reducing cognitive decline before, during, and after exercise. Recent reviews have concluded that sport plays a relevant role in neuropsychological performance (attention, information processing speed, inhibitory control, cognitive flexibility, working memory and decision-making) and that specific nutrients and diets contribute to cognitive and motor abilities. For example, vitamins (B, E, D, and C), minerals (iodine, zinc, iron, and magnesium), carbohydrates (glucose), lipids (omega-3 fatty acids), and alkaline and protein-based supplements have shown improvements, while low energy availability is associated with poor physical and cognitive performance [46]. Two studies in this Special Issue area contribute to this research area. First, Finnegan and colleagues (contribution 14) explore the knowledge of performance dietitians and nutritionists on implementing nutritional plans to support athletes with recovery from concussion (also called mild traumatic brain injuries). Second, Morton et al. (contribution 15) explore the neuroprotective effect of supplementation with a functional blend of phytonutrients containing blackcurrant, L-theanine, and pine bark extract, as well as how high-intensity interval exercise performed during ozone exposure can boost cognitive processes. Specifically, the authors analyze inflammation, Brain-Derived Neurotrophic Factor (BDNF), and neurocognition in healthy male cyclists following polyphenol supplementation and exercise in an ozone-polluted environment.

## 4. Conclusions

In summary, nutrition is a complex, crucial part of health and development that influences brain function via several networks. Adequate dietary practices help reduce neuroinflammation and oxidative stress and increase brain insulin sensitivity and the derived neurotrophic factor. Contrarily, poor-quality diets have been linked to a range of negative health outcomes, including neurological alterations that significantly contribute to long-term global disability and a lower quality of life. Multiple lifestyle factors, especially dietary patterns, have been associated with alterations in various brain systems and functions. This Special Issue provides new evidence on the impact of nutrition on various aspects of neurological function and the potential cognitive impairment associated with ED. The studies published in this issue analyze how dietary patterns and their biochemical compounds impact neuropsychological functioning. The findings provide a solid foundation for designing accurate measurement tools to detect early cognitive decline related to ED and to develop effective evidence-based interventions tailored to the specific needs of these patients.
